# A novel strategy towards efficient and reliable electric vehicle charging for the realisation of a true sustainable transportation landscape

**DOI:** 10.1038/s41598-024-53214-w

**Published:** 2024-02-08

**Authors:** B. Anil Kumar, B. Jyothi, Arvind R. Singh, Mohit Bajaj, Rajkumar Singh Rathore, Milkias Berhanu

**Affiliations:** 1 Department of Electrical and Electronics Engineering, Koneru Lakshmaiah Education Foundation, Vijayawada, India; 2 Department of Electrical Engineering, School of Physics and Electronic Engineering, Hanjiang Normal University, Hubei, Shiyan 442000 People’s Republic of China; 3 Department of Electrical Engineering, Graphic Era (Deemed to Be University), Dehradun, 248002 India; 4 Hourani Center for Applied Scientific Research, Al-Ahliyya Amman University, Amman, Jordan; 5 Graphic Era Hill University, Dehradun, 248002 India; 6 Applied Science Research Center, Applied Science Private University, Amman, 11937 Jordan; 7 Cardiff School of Technologies, Cardiff Metropolitan University, Llandaff Campus, Western Avenue, Cardiff, CF5 2YB UK; 8 Department of Electrical and Computer Engineering, Addis Ababa Science and Technology University, Adama, Ethiopia

**Keywords:** Energy science and technology, Engineering, Mathematics and computing

## Abstract

This paper proposes an innovative approach for improving the charging efficiency of electric vehicles (EVs) by combining photovoltaic (PV) systems with AC–DC Power Factor Correction (PFC). The proposed approach employs bi-directional power flow management within the PFC system, allowing for enhanced resource utilization and EV battery capacity under a variety of environmental circumstances. A modified Lyapunov-based robust model reference adaptive controller (M-LRMRAC) is developed to provide real-time Maximum Power Point Tracking (MPPT) for the PV array. By quickly recording the MPP, this controller skilfully adjusts to shifting radiation and temperature dynamics. A noteworthy accomplishment is that the M-LRMRAC outperforms traditional Perturb and Observe (P&O) techniques by achieving quick MPP convergence (0.54 s). Additionally, the benefits of this integrated system go beyond effective MPPT. The method achieves operating at unity power factor and reduces total harmonic distortion, which results in improved power quality when charging EV Batteries (EVB). The entire solution provided by this multifaceted architecture improves the quality of electricity delivered to EV batteries while also increasing energy efficiency. This research helps to the evolution of sustainable and dependable EV charging infrastructure by solving difficulties and optimising performance. The combination of PV systems with AC–DC PFC, aided by the M-LRMRAC technology, presents a viable route for attaining efficient, clean, and high-quality EV charging, hence supporting the shift to a greener and more sustainable transportation landscape.

## Introduction

Researchers in the area of EV charging have concentrated on creating effective and efficient charging systems that combine on and off-board charging^1,2^. Commonly, AC/DC and DC/DC converters are used for off-board charging. DC/DC converters which play a crucial role in voltage regulation, adapting renewable energy sources, and ensuring high efficiency to meet battery-side requirements like battery current and SOC^3,4^. Among the various DC/DC converter topologies investigated, researchers have explored different options such as DAB, DAB SRC^5^, QAB, and AQAB^6^ converters. While these converters offer galvanic isolation to achieve ZVS for all switches at the load side. The issues arise when multiple EVs are connected to a DAB converter. Balancing voltage on a dc link becomes challenging, leading to increased system size and complexity during the design process^7^. To tackle this problem, a QAB converter was suggested, comprising three bridges alongside one bridge on the MV and LV sides, offering an effective solution for multiple EV battery connections. However, relying solely on DC/DC converters presents other challenges, such as voltage flicker, increased THD, and low PF^8,9^. The varying current consumption of EV charging, particularly at initial connection and maximum charge, might result in voltage fluctuations. The electrical system may be momentarily affected by this sudden rise in current if there is a transient voltage drop at the charging station. Factors like power system impedance and battery size determine the extent and duration of this voltage drop. Significant decreases can result in voltage flicker, which is the term for flickering lights or electronic faults. If EV charging stations have poor power factor, they may incur penalties and higher losses if they are not corrected^10,11^. Additionally, the absence of harmonic filtering may lead to voltage fluctuations and potential difficulties like transformer overheating. For the wider electrical grid and the charging infrastructure to continue operating efficiently, these issues must be resolved^12^. To mitigate these issues, a novel AC/DC converter has been introduced, which not only provides PFC but also achieves a high PF^13^. There have been studies on a number of AC/DC PFC converter configurations, including buck^14^, boost^15^, and buck-boost^16^. Among them, the Ferdowsi PFC converter stands out as a suitable option for EV charging applications due to its low semiconductor count and bidirectional power flow capability^17^. The Ferdowsi PFC converter is integrated buck boost converter, can handle variations in input voltage conditions, maintain a stable DC link voltage, and achieve high efficiency while minimizing THD. Considering all these parameters related to AC/DC PFC and DC/DC converters are both, researchers go on designing off-board charging systems to efficiently charge EVB. The goal is to create a robust and reliable charging infrastructure that can support the growing demand for electric mobility while ensuring energy efficiency and stable power delivery to EVB^18,19^. Charging RV’s off-board without using solar panels faces key issues that could hinder EV popularity. Limited charging stations can make it hard to charge EVs without solar power, especially where charging infrastructure is lacking^20,21^. This also leads to concerns about running out of power on longer trips or in remote areas. Off-board charging relies on the grid, tying EVs to its capacity and energy sources^22^. If the grid uses fossil fuels, it reduces EVs’ environmental benefits due to carbon emissions. The growing number of EVs might strain the grid, needing costly upgrades and time. Another issue lies in charging costs. Off-board charging, particularly in regions where electricity prices are relatively high, might lead to increased operational expenses for EV owners, potentially impacting the overall cost-effectiveness of EV compared to traditional ICEV^23,24^. Lastly, the absence of solar PV panels means a missed opportunity to utilize renewable energy sources for charging EVs. Solar energy can be harnessed directly from the sun, providing a clean and sustainable power supply for EVs^25^. Without this renewable energy aspect, the full potential of EV’s in reducing greenhouse gas emissions and combating climate change might not be realized^26^.

Nevertheless, the frequent requirement of grid power for charging EVs presents several challenges that must be addressed^27^. The neighborhood distribution system may suffer as a result of the extensive use of EV. When many EV owners plug in their vehicles to charge simultaneously after returning home from work. The local power grid has to supply significantly more electricity as a result^28,29^. Technical concerns caused by this increase in demand may include fluctuations in frequency, harmonic disruption, and voltage regulation^30,31^. An unexpected and excessive peak load at the distribution network may result from the increasing EV usage and the regulation impact around their charging. Towards solve this problem, power utilities must upgrade the distribution grid to handle the increased demand for EV charging effectively^32,33^.

Furthermore, the distribution grid is facing a growing presence of home PV systems. During periods of lower household consumption, such as noon time and the feeder receives the extra energy produced by HPV systems^34^. Leading to voltage rise and potential line overload. To address this issue, ESU are commonly used to mitigate the impacts of high HPV system integration. In recent years, the concept of utilizing EVB as an ESS addresses the intermittency of PV systems^35^. EVBs can function as ESS, allowing them to be charged with excess solar when necessary, pv power can be used to replace the grid. By absorbing power from widespread adoption of HPV in the distribution network are made possible by EVBs' ability to reduce afternoon voltage surge concerns^36,37^. Additionally, the idea of using EVBs to support the high grid usage has been experienced also garnered increasing interest.

**Figure 1:**
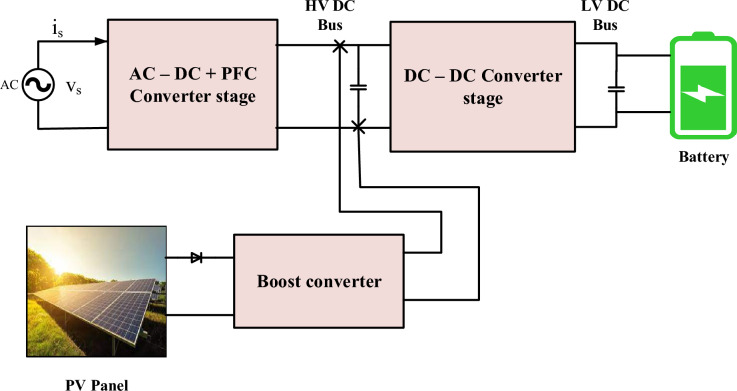
Off-board charging station with integrated PV.

So the integration of PV into the off-board charging system includes converters for both ac and dc pro vides an adequate solution as shown in Fig. 1. Moreover, integrating PV into the off-board charging system enhances energy efficiency. Traditional charging methods rely solely on the electrical grid, which may involve energy losses during transmission and conversion processes. However, with PV panels directly generating electricity on-site, there are fewer energy losses, resulting in a more efficient charging process^38,39^. This efficiency not only benefits the environment but also contributes to cost savings and optimized resource utilization. Moreover, PV integration enhances the overall energy efficiency of the charging system^40^. Traditional electricity generation and distribution involve energy losses along the way, from power plants to charging stations. By tapping into solar energy directly at the charging location, these energy losses are minimized, resulting in a more efficient charging process^41^. This increased efficiency not only benefits the environment but also optimizes the use of avail- able resources, making EV charging more sustainable in the long run. Additionally, integrating PV into off-board charging can lead to cost savings for EV owners^42^. Solar energy is essentially free once the PV infrastructure is installed, making the cost of electricity for charging significantly lower compared to relying solely on grid power. While there may be an initial investment in setting up the PV panels and related equipment, the long-term savings on energy costs can make EV ownership more economically viable and appealing to a broader range of consumers^43,44^. Furthermore, in some regions, there may be incentives or government support programs that further reduce the financial burden of PV installation, making it even more attractive for EV users^45^.

An integrated converter for EV’s that combines utility grid and solar PV charging uses a single converter for both sources, reducing component count, and employs an approach for PFC using inductor voltage detection without the need for a current sensor. The system operates seamlessly in all EV modes: charging, PRN, and RBG, functioning as a SEPIC converter for charging and as boost and buck converters for PRN and RBG modes, respectively^46,47^. Dual-source EV chargers combine grid power with solar electricity to overcome the drawbacks of solar PV-based chargers. Regardless of the solar variability or time of day, these hybrid systems guarantee dependability. In order to optimize energy use and save operating expenses, advanced energy management controls balance power sources, monitor solar generation, and integrate with the grid. This cost-effective and environmentally friendly method increases allure of electric cars by offering reliable, continuous charging services^48^.

In^49^ presents a multipurpose PEI for PEV, capable of utilizing dual charging sources encompass solar PV panels and the conventional grid supply for charging without additional components. Proposed PEI, derived from a conventional SEPIC converter, achieves MPPT during solar charging. It operates efficiently across all modes of transportation (charging, PRN, and RBG) with isolation in each mode, ensuring improved safety for batteries and vehicle users. The single converter design enhances charger compactness, making it an ideal solution to power applications that charge on board batteries.

In^50^ presents the creation of an EV charging station with solar energy. It places a strong emphasis on quiet, optimal transit using EVs and PHEVs compared to ICEVs. The study focuses on integrating renewable solar energy with the charging system. A charging circuit is included with PEV that maintains a near-unity PF through PFC and low current ripple through a PWM boost rectifier and DC/DC buck converter. By taking into account steady-state, transient, and configurable EV charging capacity responses relying upon battery SOC, a DSP controller board ensures optimal circuit operation. When it comes to connecting EVs to power sources, the CCS is essential. The vehicle's connection to the grid, which includes solar PV or home power installations, is supported by the system. In order to provide effective communication for safe and efficient charging, the CCS functions as a smart connector, facilitating a complex “handshake” between the vehicle and the power source. In order for the EV to draw electricity and for intelligent communication to optimize the charging process and guarantee that safety rules are followed, a smooth connection must be established.

In^51^ Simple integration of PQ compensating characteristics into solar panels connected to a three-phase grid. It utilizes a three-phase VSC to convert generated DC power to AC. The control approach based on an AGMVC enables effective active power transfer and PQ compensation. With the help of the P&O based MPPT algorithm, the solar PV array is used effectively. The effectiveness of the AGMVC control technique among asymmetrical load conditions is demonstrated by the fact that the grid current maintains its balance and sinusoidal shape.

In^52^ the incorporation of PV systems, traction batteries, and the AC grid is encompassed by the PEV battery charging, a centralized simultaneous multiport DC–DC converter is proposed. This converter allows both PV panels and the grid to deliver power to high-voltage batteries simultaneously or separately, making it more reliable than conventional topologies. It uses a half-bridge CLLC converter with fewer switches for bidirectional power transfer between batteries and the AC grid. The unified controller, combined with an optimum MPPT algorithm, effectively regulates the converter’s operation.

In^53^ presents a charging station utilizing solar PV, BES, DG, and grid to provide continuous charging in various modes (islanded, connected to the grid, and connected to a DG set). This facility prioritizes solar PV and BES charging for EV batteries but intelligently switches either a DG set or the grid can be connected when necessary. For achieving optimal fuel efficiency, the DG set is operated at 80–85% of its capacity. In the absence of a mechanical speed limiter, the frequency and voltage of the generator are controlled by the charging station ensures unity PF during nonlinear loading, and synchronizes PCC voltage with the grid/generator for ceaseless charging. It also facilitates active/reactive power transfer and power exchange between EVs for enhanced efficiency. In both grid-connected and islanded environments, the Charging Station solves the problems associated with single-mode operation. In islanded mode, a storage battery provides steady power despite solar variability, while in grid mode, it guarantees solar PV use even in the absence of the grid. A DG set is integrated to ensure continuity, particularly in remote places, while EV charging harmonics impact its performance. To ensure efficient functioning, the CS uses a voltage source converter to deliver reactive current and harmonics, mitigating this.

In^54^ discusses the growing utilization of EV’s worldwide and the need for self-sustainable charging stations to minimize fossil fuel consumption. It highlights the potential negative impact of fossil fuel-powered charging infrastructures on the distribution system and the environment, proposing PV energy as an efficient solution. To ensure effective grid planning and load management with a large number of EVs, centralized and decentralized control strategies should be employed, and EV load characteristics should be considered in substation planning and capacity design.

The normal operation of smart houses in an SMG is improved using an intelligent strategy that uses a broad dragonfly algorithm and logical hierarchy approach^55^. The technique reduces the peak to average consumption of smart houses while simultaneously optimizing energy sources, which is accomplished by employing solar panels and PHEVs. A system that controls the bidirectional flow of power for a solar water pumping system that is connected to the grid. To run the water pump continuously regardless of weather, it uses a without current-phase indicators, BLDC motor-drive. The system enables the utility grid to receive any extra electricity that is generated. In order to reduce switching losses, a single-phase VSC with a UVT generation approach is employed for bidirectional power flow management. UPF and reduce THD of the grid is done by the PV array at MPP^56^. The recently installed PV-based pumping system with a BLDC motor drive enhances flexibility and efficiency, addressing shortcomings of previous systems with bidirectional power flow. This invention maximizes the use of solar energy by allowing power to move from the PV array to the utility grid when water pumping is not required. On the other hand, the system permits power to flow from the grid to the BLDC motor-pump when there is insufficient PV power or at night. By ensuring continuous functioning, its bidirectional capacity overcomes the drawbacks of unidirectional systems. It provides a more adaptable and durable solution that permits the best possible energy use under a range of operating conditions, hence enhancing the PV-based pumping system's overall performance.

The synchronizing of EV charging and power allocation optimizing procedures is carried out by the two-stage system. Which involves the distribution of power based on their respective preferences, and the attainment of a balanced allocation is derived from game theory in relation to the Nash equilibrium. Moving into the subsequent phase, the confirmed overall charging capacity is presented as a fused constraint, addressing the challenge of coordinating EV charging in a distributed manner, considering individual charging requirements showing improved battery SOC, smoother grid power profile with lower peak-to-average ratio, and peak power, confirming its effectiveness^57^. In order to address power shortages at charging stations with PV and battery systems, a two-stage energy management technique is proposed. It intelligently distributes power between the grid, batteries, and solar PV in the initial stage. The second stage then makes sure that each EV receives an equitable share of the available electricity. This methodology takes into account the restricted capacity of the charging station, the constantly fluctuating demands of electric vehicles, and the sporadic nature of renewable energy sources such as solar power. By utilizing game theory, it maximizes decision-making and offers an adaptable and competitive framework to deal with power outages and improve charging operations' overall efficiency.

Atmospheric conditions, dust, temperature, cloud cover, and geographic location are just a few of the variables that might affect how much solar energy can be harvested. Due to shifting azimuth angles, the amount of solar radiation varies during the day. Even under cloudy skies, electricity production from PV panels varies depending even in the presence of constant irradiance and temperatures, generation is impacted by the panel's resistive load. Due to day-to-day fluctuations in temperature and irradiance, determining the MPP and computing it at any given load becomes challenging. A significant MPPT topology for solar cells is the use of buck-boost, boost, and buck converters to solve this problem. Due to its benefits of reduced switching losses and inductivity, which reduce current ripples, the boost converter is especially ideal for PV applications. Furthermore, a stable current with reduced stress compared to other topologies during operation is upheld by this converter. For the purpose of performance enhancement, a control algorithm is necessitated by MPPT devices. Nevertheless, suboptimal performance in MPPT applications is brought about by the PID controller's simple structure^58^. Under steady weather conditions the MPP is done by using the traditional approaches like INC^59^ and P&O^60^.

The simplicity of common MPPT methods like INC and P&O makes them widely employed. There are drawbacks to these conventional methods, though. Although their simplicity of construction makes them less weather-adaptive, they are nonetheless effective at tracking MPP in conditions of consistent weather. Because they frequently show oscillations close to the MPP traditional MPPT algorithms—like INC and P&O—are less effective for solar power plants of a larger size. When trying to maximize solar panel power extraction under dynamic weather circumstances, where these traditional approaches might not be able to provide stable and accurate tracking, this disadvantage becomes very important.

A novel approach combined to quickly get the MPP regarding fluctuating radiation and temperature, the modified MRAC using the Lyapunov and INC technique is designed. Simulations using PSIM demonstrate the robustness of the proposed control law, showcasing its superior MPP tracking capability compared to the INC-PI controller^61^. Another MPPT controller utilizing an ANFIS is presented, offering effective MPP tracking under varying environmental conditions, outperforming traditional methods like P&O and FLC. Furthermore, with great accuracy, ranging from 99.5 to 99.9%, and under a variety of radiation and temperature circumstances, an improved MPPT method based on FLC is achieved.

Despite being easy to use and producing excellent results, classic MPPT techniques have undesirable oscillations close to the MPP, according to the scant literature review. Despite their effectiveness in MPPT, soft-computing techniques are characterized by intricacy, high costs, and significant computational demands. The conventional MRAC is also not totally appropriate for some plants, such as PV systems with boost converters, which exhibit second-order system characteristics, due to its primary design for first-order systems.

A new M-LRMRAC is designed that extends the control from first order to second order and is implemented to reach MPPT in PV systems. The primary objective is to minimize system control complexity while effectively managing uncertainties and environmental disruptions. The uniqueness of this research lies in the development of an M-LRMRAC control law customized explicitly for second-order PV MPPT systems. Notable advantages of this M-LRMRAC technique include its simplicity, enhanced active adaptability, minimal Swings in close proximity to the MPP, and rapid observation rate, particularly under dynamic weather conditions.

In addition, the M-LRMRAC controller has outstanding tracking ability, capturing the MPP in variable environmental variables like temperature and sun radiation changes with an average convergence time of 0.54 s.

- A new M-LRMRAC controller is released for solar PV systems with improved MPPT.
- The simplicity, quick convergence time, quick dynamic response, and low oscillations close to the MPP define this M-LRMRAC controller.
- The MRAC controller exhibits robustness against variations in temperature and solar radiation because of its adaptive nature.
- For MPP tracking, the suggested controller performs better in terms of convergence time when compared to traditional algorithms like P&O
- The suggested controller has a convergence speed that is ten times quicker than that of conventional P&O techniques, which is noteworthy.

The organization of the remaining portion of this article is as follows: “Boost converter model” section. Boost Converter Model, “Proposed MPPT Methodology” section. Proposed MPPT methodology, “System design” section. System design, “Procedure for M-LRMRAC” section. Procedure for M-LRMRAC, “Results and discussion” section. Results and Discussion.

## Boost converter model

**Figure 2:**
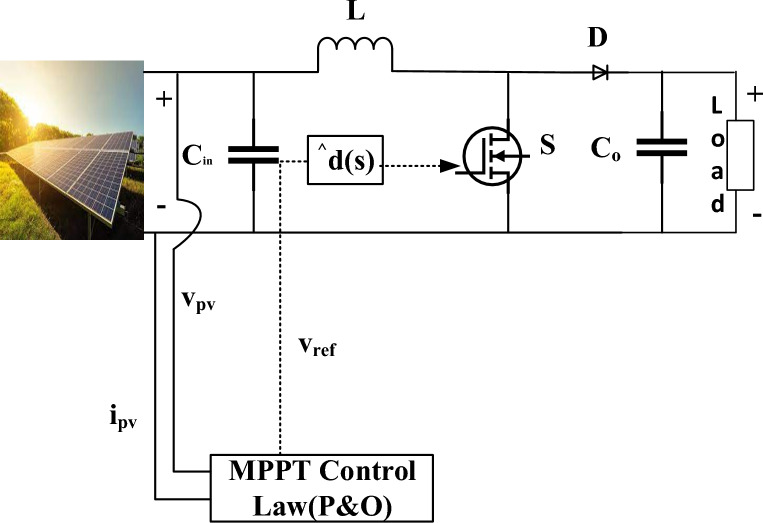
PV with Boost converter and MPPT control.

**Figure 3:**
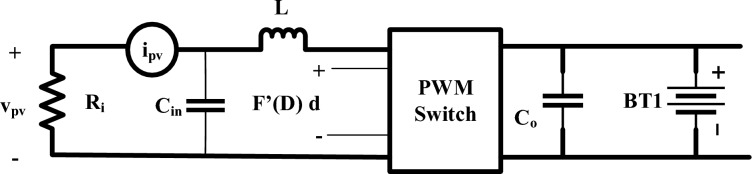
Small signal equivalent circuit of Boost converter.

The boost converter, which plays a crucial role in contemporary Optimizing the system's parts and reducing energy waste are necessary to achieve this is demonstrated in Fig. 2. Depending on specific needs, other converters might potentially be used. In the boost converter approach depicted in Fig. 3, the duty cycle (d(t)) provided to the switch S is controlled as a consequence of MPPT controller, which also continually monitors the solar array voltage and current levels is modified as necessary. This modification is achieved using Eq. 1, which establishes the connection between both the array's voltage and d(t).

1 $$v_{{{\text{pv}}}} = {\text{i}}_{{{\text{pv}}}} {\text{R}}_{{0}} (1 - {\text{d}})^{2}$$

*i*_*pv*_ and *v*_*pv*_ are commonly used notations to represent the PV array voltage and current. Every *v*_*pv*_, *i*_*pv*_ and DC *v*_*pv*_, *i*_*pv*_ components, which signify the load resistance *R*_0_. Equation (1) provides the foundation for the traditional MPPT method’s calculation of the steady-state duty cycle. Maximum transient response and suppression of oscillation with duty cycle across the connection are controlled by MPPT. Performance under a variety of various environmental situations is poor due to this lack of adaptation.

The presentation and discussion of a detailed boost converter transient analysis are carried out. Toward improve every comprehensibility about transient response study, it is believed that a small-signal equivalent circuit can represent the system, as discussed in^62^. Figure 3 illustrates the linearized PV model which utilizes resistor R_i_ and small-signal representations of the *v*_*pv*_ and *i*_*pv*_ across its terminals to effectively model the solar array.

Currently, the control signal (d(t)) actively regulates beyond the array voltage through the TF relationship at the supplied operating point. This TF represents dynamic of the system. A dynamic model showing a typical PV system battery load is shown in Fig. 3. Here, ignoring battery dynamics in the process manner in which fluctuations are the duty cycle of a system are brought about by variations in voltage across the array is revealed by the transfer function. Following correlation^63^ is obtained from this analysis of Fig. 3 and presented in Eqs. 2 and 3.

2 $$\frac{{{\hat{\text{v}}}_{{{\text{pv}}}} \left( {\text{s}} \right)}}{{{\text{R}}_{{\text{i}}} }} + {\text{s}\hat{v}}_{{{\text{pv}}}} \left( {\text{s}} \right){\text{C}}_{{{\text{in}}}} = ({\text{F}}^{\prime } \left( {\text{D}} \right){\hat{\text{d}}}\left( {\text{s}} \right) - \frac{{{\hat{\text{v}}}_{{{\text{pv}}}} \left( {\text{s}} \right)}}{{{\text{sL}}}}$$

The derivative of *F*′(*D*) with respect to D is given in the following Eq. 3

3 $$\frac{{{\hat{\text{v}}}_{{{\text{pv}}}} \left( {\text{s}} \right)}}{{{\hat{\text{d}}\text{s}} }} = \frac{{{\text{F}}^{\prime } \left( {\text{D}} \right)}}{{{\text{s}}^{{2}} {\text{LC}}_{{{\text{in}}}} + \frac{{\text{L}}}{{{\text{R}}_{{\text{i}}} }} {\text{i}}_{{\text{s}}} + 1}}$$

knows that F(d) in below Eq. 4

4 $$F\left( D \right) = V_{{{\text{PV}}}} = \left( {1 - D} \right)V$$

From Eq. 4*F*′(D) presented in Eq. 5

5 $$\frac{{{\hat{\text{v}}}_{{{\text{pv}}}} \left( {\text{s}} \right)}}{{{\hat{\text{d}}\text{s}} }} = \frac{{\frac{{ - {\text{V}}_{{\text{o}}} }}{{{\text{LC}}_{{{\text{in}}}} }}}}{{{\text{s}}^{{2}} {\text{LC}}_{{{\text{in}}}} + \frac{1}{{{\text{R}}_{{\text{i}}} {\text{C}}_{{{\text{in}}}} }}{\text{i}}_{{\text{s}}} + \frac{1}{{{\text{LC}}_{{{\text{in}}}} }}}}$$

## Proposed MPPT methodology

In the framework of a second-order type PV MPPT system, the proposed study’s goal is to develop an M-LMRAC control rule that makes use of the Lyapunov stability theorem. Improving the efficiency of the MPPT algorithm for a second-order PV system is the primary goal of the proposed study. The main goals are to overcome the shortcomings of traditional methods and to increase overall efficiency, tracking speed, accuracy, and complexity in the face of changing environmental conditions. The second-order extension of the suggested adaptive MRAC architecture seeks to reduce system complexity and efficiently handle environmental and PV system uncertainties and disturbances. By developing an M-LMRAC control method using the Lyapunov stability theorem, the paper presents a novel strategy for improving MPPT in second-order PV systems.

**Figure 4:**
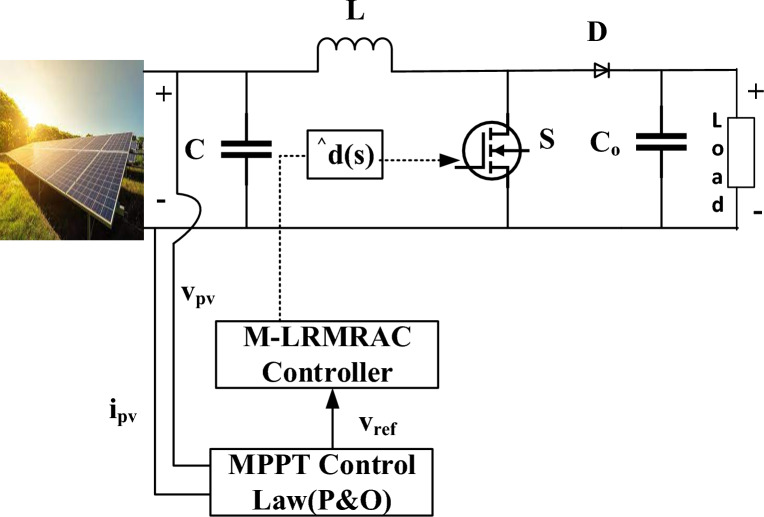
M-LRMRAC controller with Solar Boost converter.

Figure 4 comprising of an initial MPPT control block succeeded by a subsequent M-LRMRAC module, showcasing a novel configuration represented consecutive two-tier hybrid strategy is encompassed by the proposed MPPT approach. In the first level, a *v*_*ref*_ for each MPPT is generated using the conventional P&O approach. Subsequently, this MPP voltage is updated continuously and compared to the varying *v*_*pv*_ resulting from fluctuations in thermal conditions, electric demand, irradiation. The M-LRMRAC controller's input is created by the difference between v_pv_ and the adjusted v_ref_ and key challenge lies in determining suitable controller parameters for achieving effective performance. The suggested system parameters are refined throughout the adaptation process by exploiting the difference between the referring to both the controller and the system models. Using appropriate adaptive principles. Closed-loop stability is ensured, and the adaptive rules have the capability directed toward forecasting the undisclosed parameters of the controller. Using M-LRMRAC to serve as an initial signal for PWM duty cycle modification within the boost converter configuration, the Lyapunov stability theorem is implemented to actively maintain the PV panel at its MPP.

Using the formulation supplied in Eq. 6, a tailored v_ref_ variation is determined for the controller, allowing the MPPT regulation law to effectively pinpoint the precise operational junction for best power extraction, where ∆*v* represents a small threshold voltage, and *v*_*pv*_.

### MPPT control block (P&O)

Equation 6 depicts the connection that governs the variance of *v*_*ref*_ that corresponds with the P&O MPPT approach, ensuring optimal tracking of the MPP of the solar panel.

6 $${\text{v}}_{{{\text{ref}}}} = \left\{ {{\text{v}}_{{{\text{pv}}}} , \frac{{d_{p} }}{{d_{pv} }} = 0, \quad {\text{v}}_{{{\text{pv}}}} , \frac{{d_{p} }}{{d_{pv} }} < 0,\quad {\text{v}}_{{{\text{pv}}}} , \frac{{d_{p} }}{{d_{pv} }} > 0} \right.$$

### Proposed M-LRMRAC approach

A responsive control mechanism is required, which can quickly adapt the duty cycle to be aligned with rapidly changing environmental factors, ensuring the optimal operation of the PV panel at its MPP, even when there are abrupt fluctuations in solar insolation, for instance shifting sunlight intensities or impacts caused by shading. A substantial preference for second-order dynamics is noted in the field of plant features, which includes a variety of systems including PV installations with boost converters. Despite their widespread adoption, traditional MRAC approaches fall short of offering sufficient tracking performance for second-order systems.

**Figure 5:**
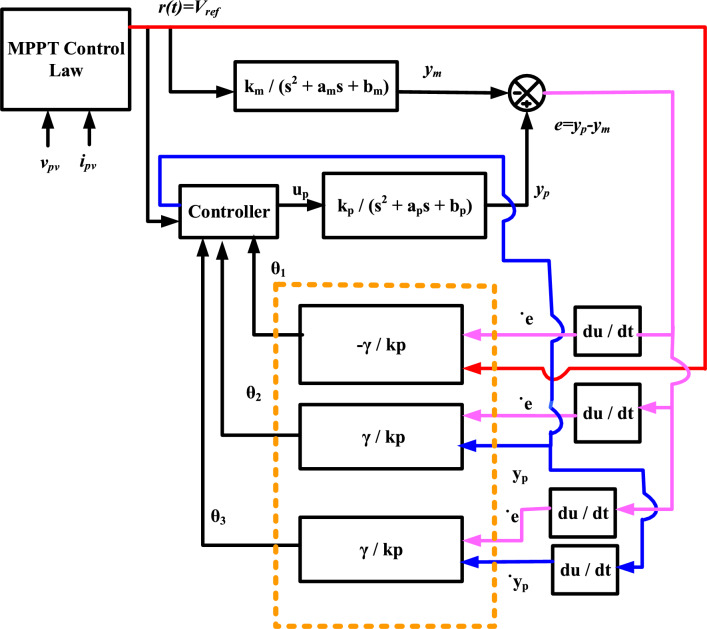
Design of M-LRMRAC controller.

As a result, this study seeks to get around these limitations by developing a novel control law that is deliberately designed to solve second-order system complexities. This breakthrough results in the development of a M-LRMRAC strategy that bridges the gap between first and second-order dynamics. Figure 5 depicts a visual representation of the suggested M-LRMRAC idea, with the signal r(t) playing a critical role. Equation 5’s transfer function precisely matches the plant model depicted in Fig. 5.

Here, the plant’s output and input are represented by y_p_(t) and u_p_(t), respectively.

7 $$\frac{{{\text{d}}^{{2}} {\text{y}}_{{\text{p}}} \left( {\text{t}} \right)}}{{{\text{dt}}^{{2}} }} = - {\text{a}}_{{\text{p}}} \frac{{{\text{dy}}_{{\text{p}}} \left( {\text{t}} \right)}}{{{\text{dt}}}} - {\text{b}}_{{\text{p}}} \frac{{{\text{dy}}_{{\text{p}}} \left( {\text{t}} \right)}}{{{\text{dt}}}} + {\text{k}}_{{\text{p}}} {\text{u}}_{{\text{p}}} \left( t \right)$$

8 $${\text{G}}_{{\text{p}}} \left( {\text{s}} \right) = \frac{{{\text{y}}_{{\text{p}}} \left( {\text{s}} \right)}}{{{\text{u}}_{{\text{p}}} \left( {\text{s}} \right)}} = \frac{{{\text{k}}_{{\text{p}}} }}{{{\text{s}}^{{2}} + {\text{a}}_{{\text{p}}} {\text{s}} + {\text{b}}_{{\text{p}}} }}$$

For desired second order reference model along with plant, presented in the Eq. 9

9 $$\frac{{{\text{d}}^{{2}} {\text{y}}_{{\text{m}}} \left( t \right)}}{{{\text{dt}}^{{2}} }} = - {\text{a}}_{{\text{m}}} \frac{{{\text{dy}}_{{\text{m}}} \left( {\text{t}} \right)}}{{{\text{dt}}}} - {\text{b}}_{{\text{m}}} \frac{{{\text{dy}}_{{\text{m}}} \left( t \right)}}{{{\text{dt}}}} + {\text{k}}_{{\text{m}}} {\text{r}}\left( {\text{t}} \right)$$

10 $${\text{G}}_{{\text{m}}} \left( {\text{s}} \right) = \frac{{{\text{y}}_{{\text{m}}} \left( {\text{s}} \right)}}{{{\text{u}}_{{\text{m}}} \left( {\text{s}} \right)}} = \frac{{{\text{k}}_{{\text{m}}} }}{{{\text{s}}^{{2}} + {\text{a}}_{{\text{m}}} {\text{s}} + {\text{b}}_{{\text{m}}} }}$$

The controller is depicted in following Eq. 10

11 $$u_{{\text{p}}} = \uptheta _{1} {\text{r}} - \uptheta _{2} {\text{y}}_{{\text{p}}} - \uptheta _{3} \cdot {\text{y}}_{{\text{p}}} =\uptheta ^{{\text{T}}}$$

Substitute Eq. 11 into the Eq. 7 will get the Eq. 12.

12 $$\frac{{{\text{d}}^{2} {\text{y}}_{{\text{p}}} \left( {\text{t}} \right)}}{{{\text{dt}}^{2} }} = - ({\text{a}}_{{\text{p}}} + {\text{k}}_{{\text{p}}}\uptheta _{3} )\frac{{{\text{dy}}_{{\text{p}}} \left( {\text{t}} \right)}}{{{\text{dt}}}} - ({\text{b}}_{{\text{p}}} + {\text{k}}_{{\text{p}}}\uptheta _{2} ){\text{y}}_{{\text{p}}} ({\text{t}}) + {\text{k}}_{{\text{p}}}\uptheta _{1} {\text{r}}({\text{t}})$$

Compare the Eqs. 12 and 9 will get the controlled parameters of plant in terms θ_1_, θ_2_, θ_3_

13 $$\uptheta _{1} = \frac{{{\text{k}}_{{\text{m}}} }}{{{\text{k}}_{{\text{p}}} }}$$

14 $$\uptheta _{2} = \frac{{{\text{b}}_{{\text{m}}} - {\text{b}}_{{\text{p}}} }}{{{\text{k}}_{{\text{p}}} }}$$

15 $$\uptheta _{2} = \frac{{{\text{a}}_{{\text{m}}} - {\text{a}}_{{\text{p}}} }}{{{\text{k}}_{{\text{p}}} }}$$

The eror from the plant controller and V_ref_ will be the

16 $$e = {\text{y}}_{{\text{p}}} - {\text{y}}_{{\text{m}}}$$

Double derivative of Eq. 16 with respect to t will give the Eq. 17

17 $$\frac{{{\text{d}}^{{2}} {\text{e}}\left( {\text{t}} \right)}}{{{\text{dt}}^{{2}} }} = - ({\text{a}}_{{\text{p}}} + {\text{k}}_{{\text{p}}}\uptheta _{3} )\frac{{{\text{dy}}_{{\text{p}}} \left( {\text{t}} \right)}}{{{\text{dt}}}} - ({\text{b}}_{{\text{p}}} + {\text{k}}_{{\text{p}}}\uptheta _{2} ) {\text{y}}_{{\text{p}}} ({\text{t}}) + {\text{k}}_{{\text{p}}}\uptheta _{1} {\text{r}}({\text{t}}) + {\text{a}}_{{\text{m}}} \frac{{{\text{dy}}_{{\text{m}}} \left( {\text{t}} \right)}}{{{\text{dt}}}} + {\text{b}}_{{\text{m}}} \frac{{{\text{dy}}_{{\text{m}}} \left( {\text{t}} \right)}}{{{\text{dt}}}} - {\text{k}}_{{\text{m}}} {\text{r}}\left( t \right)$$

## System design

### Estimation of PV power rating

In the research process, the PV array parameters, including the number of panels required, MPPT mechanism, and Boost converter specifications, will be designed based on the EVB specifications. The power rating of the PV array can be identified using three EVBs, each with an operational voltage and current of 48 V and 25 A, respectively.

*Step 1:*

- Determine total charge power required for the three batteries:
- Total Charge Power (W) = Total Voltage (V) x Charge Current (A).
- Total Voltage = 3 × Maximum V_b_ = 3 × 48 V = 144 V.
- Charge Current = 25 A.
- Total Charge Power (W) = 144 V × 25A = 3600 W.

*Step 2:*

- Consider the efficiency of the charging process. Assume a typical efficiency of around 80% for the entire charging system.

*Step 3:*

- Calculate the power rating of the PV panels:
- Power Rating of PV Panels (W) = Total Charge Power/Charging Efficiency.
- Power Rating of PV Panels (W) = 3600W/0.8 ≈ 4500W.

### Number of panels

Number of panels is established by a total power rating of 4500 W, will use the specifications of the Risen-335-WH solar panel, which has a power rating of 335 W, a voltage at MPP of 37.65 V, and a current at MPP of 8.90 A. Considering standard irradiation and temperature conditions of 1000 W/m^2^ and 25 °C, will design a PV array to meet the required capacity.

Number of panels needed = Total required power/Power rating of each panel.

Number of panels needed = $\frac{4500w}{{335w}} = 13.43 = 14$

But for suitable panels selection based on the series and parallel configuration will choose 15 panels. In which each row, 5 panels are connected in series. This will increase the voltage while keeping the current the same. Then, these three rows are connected in parallel. This will maintain the voltage at the level achieved by connecting 5 panels in series while increasing the total current by combining the currents of the three parallel rows.

### Design of boost converter

As part of the design procedure, the sizing of the L in the boost converter is chosen in order to sustain CCM functioning under a broad range of weather scenarios along duty ratio D_1_.

18 $${\text{D}} = \frac{{{\text{V}}_{{{\text{dc}}}} - {\text{V}}_{{{\text{pv}}}} }}{{{\text{V}}_{{{\text{dc}}}} }} = \frac{300 - 188.25}{{300}} = 0.3725$$

V_pv_ is selected as 5 times of voltage at MPP. The input inductor estimated as

19 $$L = \frac{{D_{1} V_{{{\text{pv}}}} }}{{f_{{{\text{sw}}}} \delta I_{{\text{L}}} }} = \frac{0.3725*188.25}{{50000*0.2*8.90}} = 788\;\upmu {\text{H}}$$

## Procedure for M-LRMRAC

The Lyapunov stability theorem is a fundamental concept in control theory used to analyze the stability of dynamical systems. When the framework is classified to exhibit asymptotic stability by the existence of a continuously differentiable real scalar function V(t) and its time derivative V(t) is consistently negative definite (V(t) 0) for all values of t larger than zero.

Based on the idea of utilizing a Lyapunov function to assess the stability of a dynamical system, the Lyapunov stability theorem is presented in the study. An statement in mathematics that aids in evaluating the behavior of a system over time is called a Lyapunov function. A system must have a continuously decreasing derivative of a potential energy-like variable to be deemed “asymptotically stable,” which can be achieved by using a Lyapunov function. A system is said to be “asymptotically stable” if it naturally tends to approach a stable equilibrium point over time, which is identified by a minimum or zero value of the Lyapunov function.

As for the Lyapunov function *V*(*t*) in this specific case, it is defined as follows steps:

*Step-1:*

20 $$V\left( t \right) = 0.5 e^{2} \left( {\text{t}} \right)$$

*Step-2:*

21 $$V^{ \cdot } { }\left( t \right) = \frac{d }{{dt}}\left[ {0.5e^{2} \left( t \right)} \right]$$

*Step-3:*

22 $$V^{ \cdot } \left( t \right) = e\left( {\text{t}} \right) *e^{ \cdot } \left( t \right)$$

*Step-4:*

From Eq. 16 calculate *e*˙(*t*)

23 $$e^{ \cdot } \left( t \right) = y^{ \cdot }_{{\text{p}}} \left( t \right) - y^{ \cdot }_{{\text{m}}} \left( t \right)$$

*Step-5:*

Substitute Eqs. 23 in 22 will gives the

24 $$V^{ \cdot } \left( t \right) = e\left( {\text{t}} \right) * y^{ \cdot }_{{\text{p}}} \left( t \right) - y^{ \cdot } {\text{m}}\left( t \right)$$

*Step-6:*

Determine e(t)’s second order type derivative in relation to time “t” from Eq. 23

25 $$\frac{{{\text{d}}^{{2}} {\text{e}}\left( {\text{t}} \right)}}{{{\text{dt}}^{{2}} }} = y^{ \cdot \cdot } p\left( t \right) - y^{ \cdot \cdot } m\left( t \right)$$

*Step-7:*

From Eqs. 25 and 24 the

26 $$V^{ \cdot } \left( t \right) = y^{ \cdot }_{{\text{p}}} \left( t \right) - y^{ \cdot } {\text{m}}\left( t \right) * ( - {\text{a}}_{{\text{p}}} - {\text{k}}_{{\text{p}}}\uptheta _{3} )\frac{{{\text{dy}}_{{\text{p}}} \left( {\text{t}} \right)}}{{{\text{dt}}}} - ({\text{b}}_{{\text{p}}} + {\text{k}}_{{\text{p}}}\uptheta _{2} ){\text{y}}_{{\text{p}}} ({\text{t}}) + {\text{k}}_{{\text{p}}}\uptheta _{1} {\text{r}}({\text{t}}) + {\text{a}}_{{\text{m}}} \frac{{{\text{dy}}_{{\text{m}}} \left( {\text{t}} \right)}}{{{\text{dt}}}} + {\text{b}}_{{\text{m}}} \frac{{{\text{dy}}_{{\text{m}}} \left( {\text{t}} \right)}}{{{\text{dt}}}} - {\text{k}}_{{\text{m}}} {\text{r}}\left( {\text{t}} \right)$$

Assuming *k*_*p*_ γ > 0, let’s define the Lyapunov function *V* based on the given Eq. 26 will set to V’(t) = − γ. The Lyapunov function *V* is typically for any t > 0, the system is consistent because V(t) is negative definite (V(t) 0). If V(t) equals 0 for every t > 0 in addition system is equilibrium state and any errors or disturbances will converge towards zero over time. By carefully designing the parameters *k*_*p*_, γ, and the function *V*(*t*), the control system can be made robust and stable. The Lyapunov function *V* is an essential tool in control theory to prove stability properties of a system and design suitable controllers for desired performance. Based on Eq. 26

1. $\left( {y_{p} \left( t \right) - y_{m} \left( t \right)} \right)( - {\text{a}}_{{\text{p}}} - {\text{k}}_{{\text{p}}}\uptheta _{3} )\frac{{{\text{dy}}_{{\text{p}}} \left( {\text{t}} \right)}}{{{\text{dt}}}}$ To make this term negative, present a ${\text{y}}_{{\text{p}}} \left( t \right) < {\text{y}}_{{\text{m}}} \left( t \right)$ and both $a_{{\text{p}}} \;{\text{and}}\;k_{{\text{p}}} \theta_{3}$ to be positive.
2. $\left( {y_{p} \left( t \right) - y_{m} \left( t \right)} \right)( - {\text{b}}_{{\text{p}}} - {\text{k}}_{{\text{p}}}\uptheta _{2} ) {\text{y}}_{{\text{p}}} ({\text{t}})$ To make this term negative, present a $y_{{\text{p}}} \left( t \right) < y_{{\text{m}}} \left( t \right)$ and both $b_{{\text{p}}} \;{\text{and}}\;k_{{\text{p}}} \theta_{2}$ to be positive.
3. $\left( {y_{p} \left( t \right) - y_{m} \left( t \right)} \right) \left( {{\text{k}}_{{\text{p}}}\uptheta _{1} {\text{r}}({\text{t}}) + {\text{a}}_{{\text{m}}} \frac{{{\text{dy}}_{m} \left( {\text{t}} \right)}}{{{\text{dt}}}} + {\text{b}}_{{\text{m}}} \frac{{{\text{dy}}_{{\text{m}}} \left( {\text{t}} \right)}}{{{\text{dt}}}} - {\text{k}}_{m} {\text{r}}\left( {\text{t}} \right)} \right)$ To make this term negative, present a $y_{{\text{p}}} \left( t \right) < y_{{\text{m}}} \left( t \right)$ and the coefficients $a_{{\text{m}}} , b_{{\text{m}}} , k_{{\text{m}}} , k_{{\text{p}}} \theta_{1} ,$ to be such that the entire expression in the brackets becomes negative.

The derivatives of the controller parameters with respect to time $\frac{{{\text{d}}\uptheta _{1} }}{dt}$, $\frac{{{\text{d}}\uptheta _{2} }}{dt}$, $\frac{{{\text{d}}\uptheta _{3} }}{dt}$ can be found by setting the coefficients of each term to − γ.

- 1. For the term $\left( {y_{p} \left( t \right) - y_{m} \left( t \right)} \right)( - {\text{a}}_{{\text{p}}} - {\text{k}}_{{\text{p}}}\uptheta _{3} )\frac{{{\text{dy}}_{{\text{p}}} \left( {\text{t}} \right)}}{{{\text{dt}}}}$: $-\upgamma = - \left( {{\text{a}}_{{\text{p}}} + {\text{k}}_{{\text{p}}}\uptheta _{3} } \right)$_._

Therefore $$\frac{{{\text{d}}\uptheta _{3} }}{dt} = \frac{{\upgamma - {\text{a}}_{{\text{p}}} }}{{{\text{k}}_{{\text{p}}} }}$$

- 2. For the term $\left( {y_{p} \left( t \right) - y_{m} \left( t \right)} \right)( - {\text{b}}_{{\text{p}}} - {\text{k}}_{{\text{p}}}\uptheta _{2} ){\text{y}}_{{\text{p}}} ({\text{t}})$: $-\upgamma = - \left( {{\text{b}}_{{\text{p}}} + {\text{k}}_{{\text{p}}}\uptheta _{2} } \right)$_._

Therefore $$\frac{{{\text{d}}\uptheta _{2} }}{dt} = \frac{{\upgamma - {\text{b}}_{{\text{p}}} }}{{{\text{k}}_{{\text{p}}} }}$$

- 3. For the term, $\left( {y_{p} \left( t \right) - y_{m} \left( t \right)} \right) \left( {{\text{k}}_{{\text{p}}}\uptheta _{1} {\text{r}}({\text{t}}) + {\text{a}}_{{\text{m}}} \frac{{{\text{dy}}_{{\text{m}}} \left( {\text{t}} \right)}}{{{\text{dt}}}} + {\text{b}}_{{\text{m}}} \frac{{{\text{dy}}_{{\text{m}}} \left( t \right)}}{{{\text{dt}}}} - {\text{k}}_{{\text{m}}} {\text{r}}\left( {\text{t}} \right)} \right): = -\upgamma$

Therefore $$\frac{{{\text{d}}\uptheta _{1} }}{dt} = \frac{{ -\upgamma }}{{{\text{k}}_{{\text{p}}} }}$$

The updated controlled parameters are defined in below and presented in Fig. 5:

27 $$\frac{{{\text{d}}\uptheta _{1} }}{dt} = \frac{{ -\upgamma }}{{{\text{k}}_{{\text{p}}} }}$$

28 $$\frac{{{\text{d}}\uptheta _{2} }}{dt} = \frac{{\upgamma - {\text{b}}_{{\text{p}}} }}{{{\text{k}}_{{\text{p}}} }}$$

29 $$\frac{{{\text{d}}\uptheta _{3} }}{dt} = \frac{{\upgamma - {\text{a}}_{{\text{p}}} }}{{{\text{k}}_{{\text{p}}} }}$$

## Results and discussion

**Figure 6:**
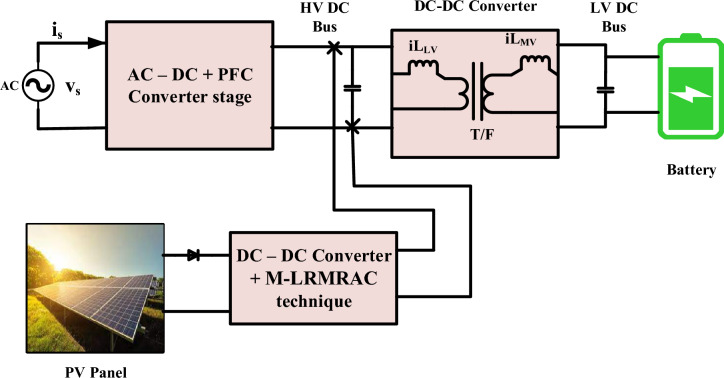
Detailed description of PV with PFC fed to EVB.

Following the integration of solar PV into AC–DC PFC and DC–DC converter fed into an EVB, Fig. 1 displays a detailed Fig. 6.

**Figure 7:**
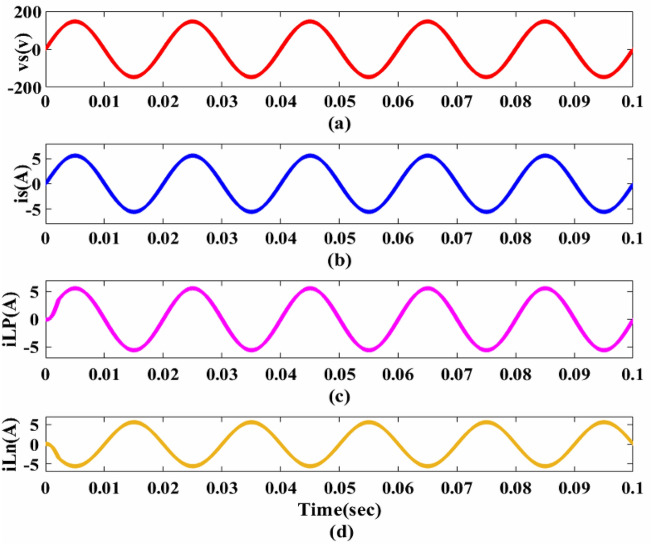
Performance of PFC: (**a**,**b**) v_s_ and i_s_. (**c**,**d**) Inductor currents iL_p_, iL_n_.

The remaining data parameters are presented in Table 1 and are based on the design specifications. This research focuses on solar PV arrays with M-LRMRAC (Fig. 7).

**Table 1:** Simulation results.

Parameter	Value
*P*_*max*_	335w
*v*_*pv*_* at P*_*max*_	37.65 V
*i*_*pv*_* at P*_*max*_	8.90 A
*v*_*oc*_	45.90 V
*I*_*sc*_	9.40 A
*v*_*s*_	220 V
*f*_*s*_	50 kHz
*v*_*b*_	48 V
*i*_*b*_	25A
C_in_	2.364 μF
L	788 μH
${\text{a}}_{{\text{p}}} = \frac{1}{{{\text{R}}_{{\text{i}}} {\text{C}}_{{{\text{in}}}} }}$	100 × 10^3^ rad/s
a_m_	8.17 × 10^3^ (rad/s)^2^
$b_{{\text{p}}} = \frac{1}{{{\text{LC}}_{{{\text{in}}}} }}$	5.368 × 10^8^ (rad/s)^2^
b_m_	1.67 × 10^7^ (rad/s)^2^
$k_{{\text{p}}} = \frac{{v_{{\text{o}}} }}{{{\text{LC}}_{{{\text{in}}}} }}$	257.664 × 10^8^ (rad/s)^2^
k_m_	5.75 × 10^8^ V(rad/s)^2^
N_se_, N_pe_	5 & 8
${\Gamma }$	0.08

Two type of analysis are performed.

1.PV + AC–DC + PFC + DC–DC fed to EVB.

2.PV + PFC + DC–DC fed to EVB.

### PV + AC–DC + PFC + DC–DC fed to EVB

**Figure 8:**
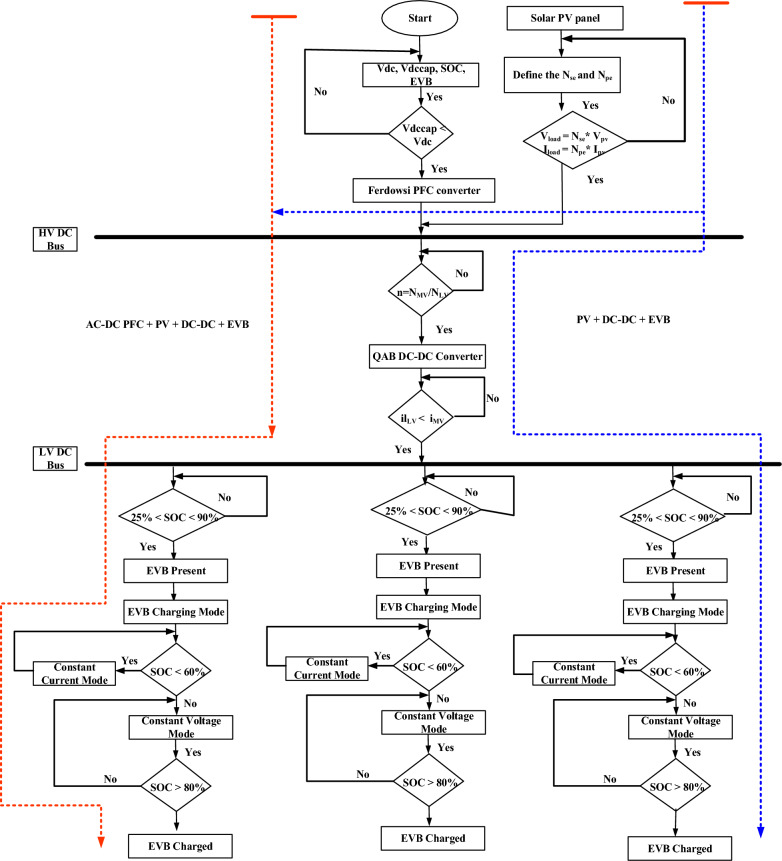
Flowchart of PV + AC/DC PFC + DC − DC + EVB.

Figure 8 shows the overall flowchart of PV + AC–DC + PFC + DC–DC fed to EV Battery. It is to be operated based on the *v*_*s*_, 220 V, and the AC–DC PFC Converter performance, demonstrating that the converter is functioning at UPF when the *i*_*s*_* peak* is in phase with *v*_*s*_* peak* as depicted in Fig. 7. Figure 7c,d indicate converter’s operation in DCM due to transition, yet the inductor currents *i*_*Lp*_ and *i*_*Ln*_ remain continuous. This continuity results from the discharge of energy stored in inductors *L*_*p*_ and *L*_*n*_, effectively transferred to output capacitor *C*_*f*_.

The inductor L_P_ releases stored energy when the switch S_P_ is open during the DCM transition, ensuring continuous current flow to the load through established inductor currents (i_Lp_ and i_Ln_). The diode D_P_, which is forward-biased, stops current backflow. Concurrently, the capacitor C_P_ maintains system stability by offering a steady voltage supply when the system is turned off. By coordinating their efforts, the load and output capacitor C_o_. will always receive power, and the converter will remain stable during the input voltage's negative half-cycle.

**Figure 9:**
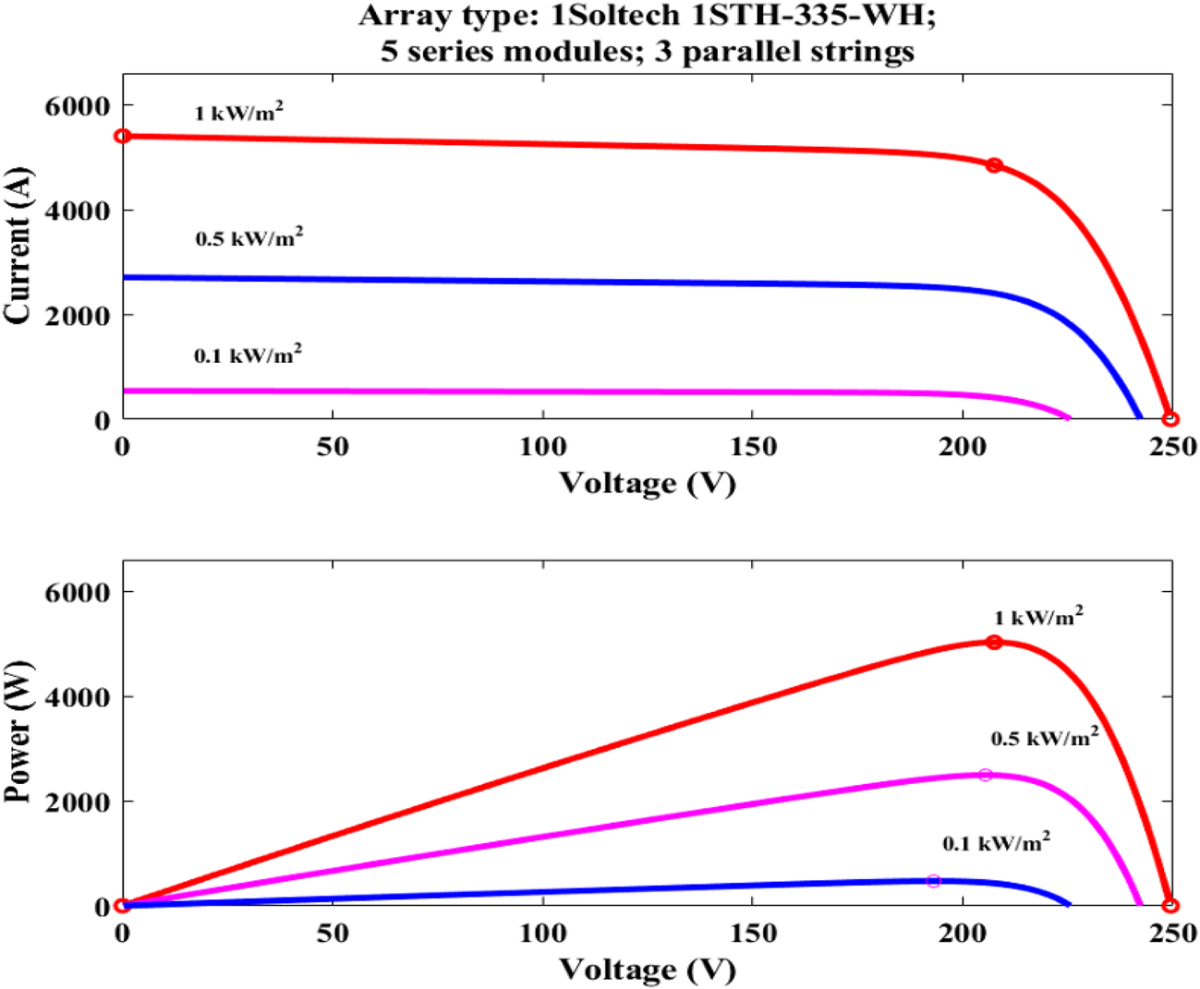
MPPT at 1000 w/m^2^.

**Figure 10:**
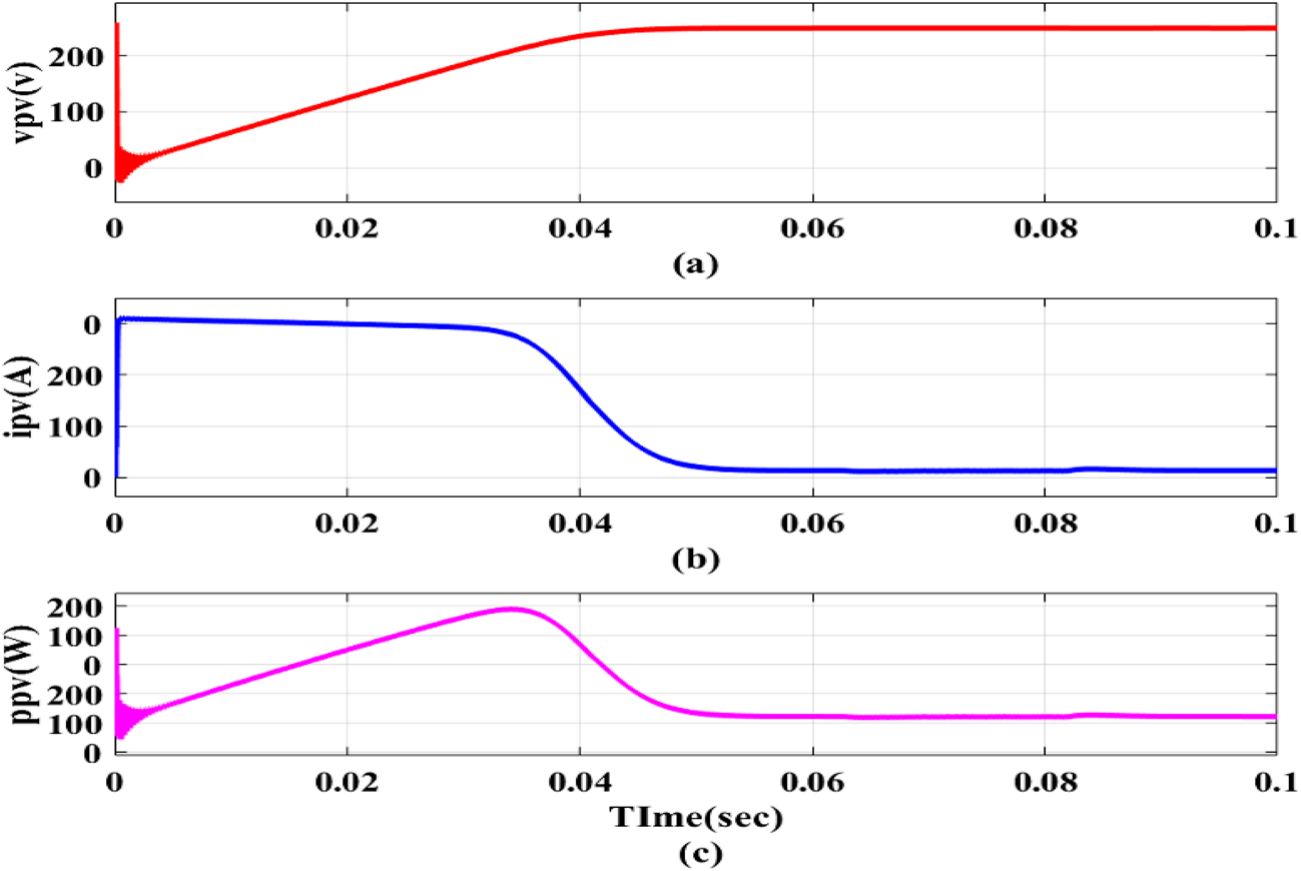
PV panel outputs.

Additionally, the PV will be connected to the PFC converter’s DC connection and will be able to run MPPTs of 1000 w/m^2^ and PV panel outputs, as indicated in Figs. 9 and 10. Depending on how the panel circuit is configured, the *i*_*p*_*v* and *v*_*p*_*v* are reduced and increased until they reach the final steady state value shown in Table 1 as indicated in Fig. 10, can generate a maximum power of 4.5 kW. An essential part of EV charging stations that facilitates effective power transfer between the EVB and the grid is a bidirectional DC–DC converter. It functions as a connection point for various DC voltage sources and loads, enabling power to flow in both ways.

**Figure 11:**
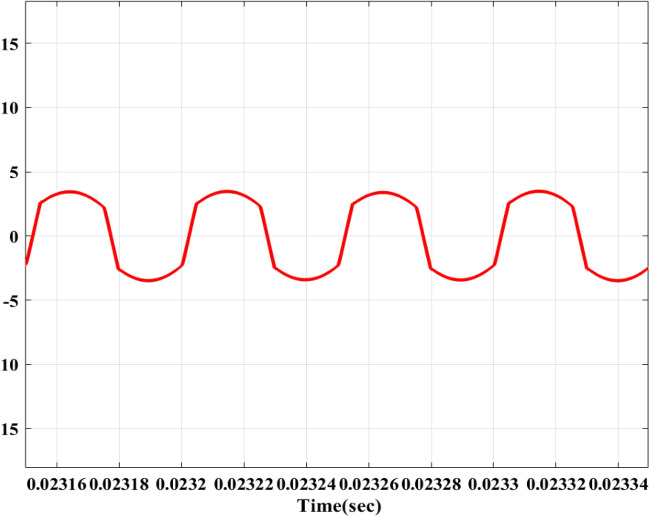
DC–DC converter triangular currents.

**Figure 12:**
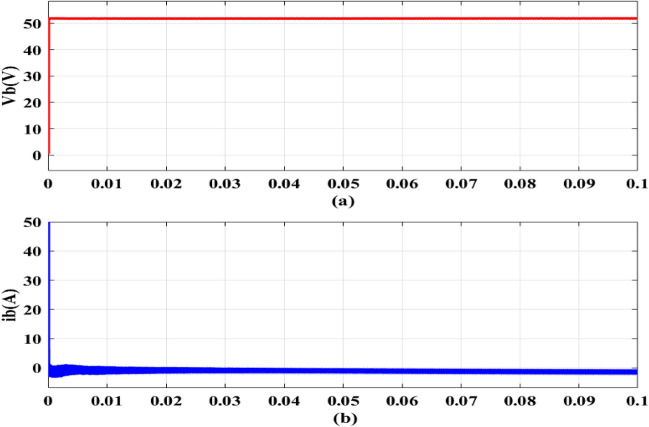
Battery outputs (**a**) v_bchar_ (**b**) i_bchar_.

For a number of applications in the EV ecosystem and the charging infrastructure, this bidirectional functionality is crucial. Circulating currents develop when a DC–DC converter operates outside of its nominal voltage conversion ratio. As a result of applying a ZVS for DC–DC converter to mitigate circulating currents, the trapezoidal current seen in Fig. 11. Figure 12a,b depict total system fed to the 48 V/100 Ah Battery. Figure 12a shows that the *V*_*bchar*_ is 51.93 V, suggesting that the *v*_*b*_ will be raised to 51.93 V while charging. EVB is capable of being charged at a maximum of 25.06 A, in accordance with an I_bchar_ value of -25.06A, verifying the expected inward current flow through this phase as indicated by the negative sign before the current measurement.

### PV + DC–DC fed to EVB

**Figure 13:**
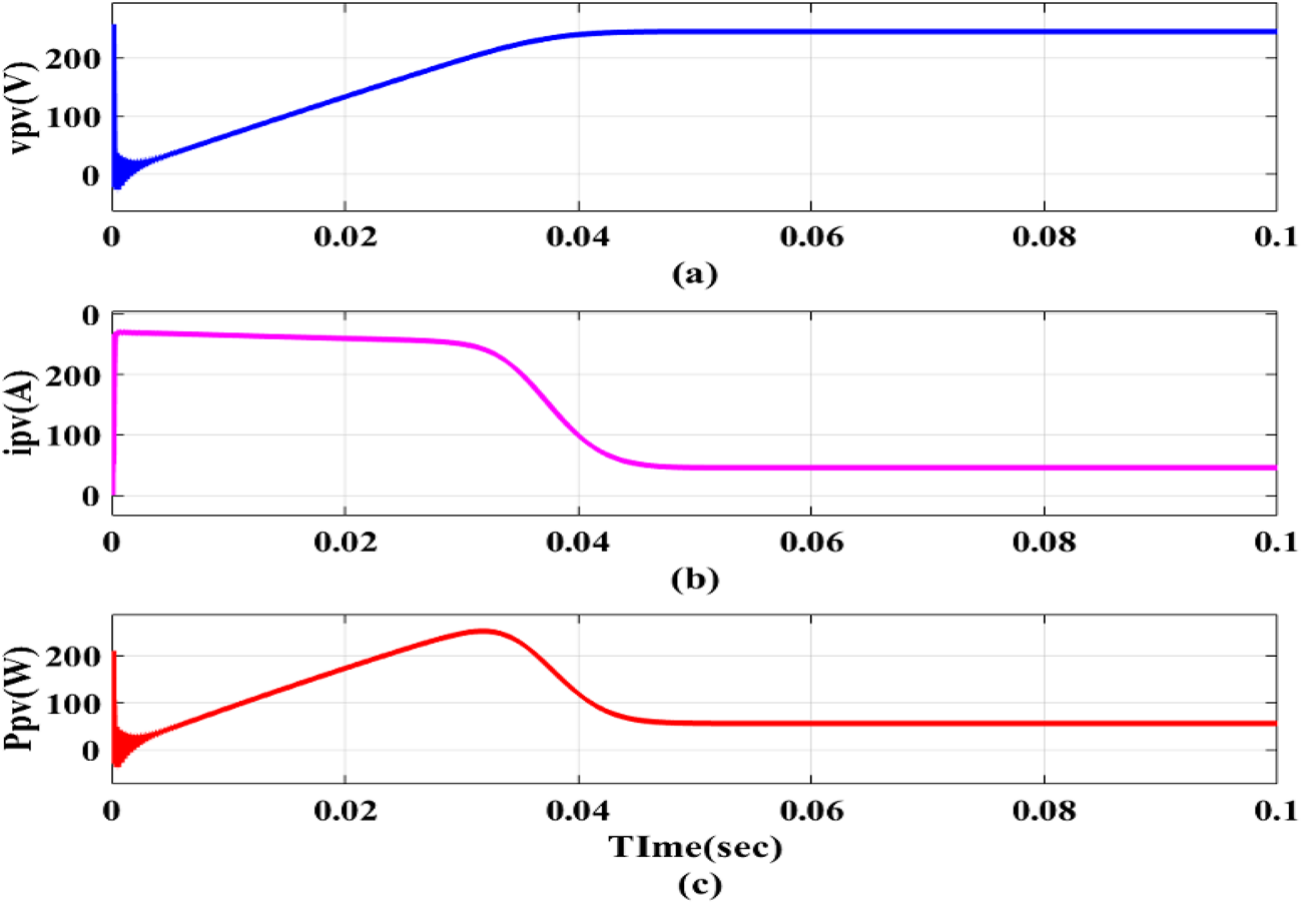
PV Outputs (**a**) v_pv_ (**b**) i_pv_ (**c**) P_pv_.

**Figure 14:**
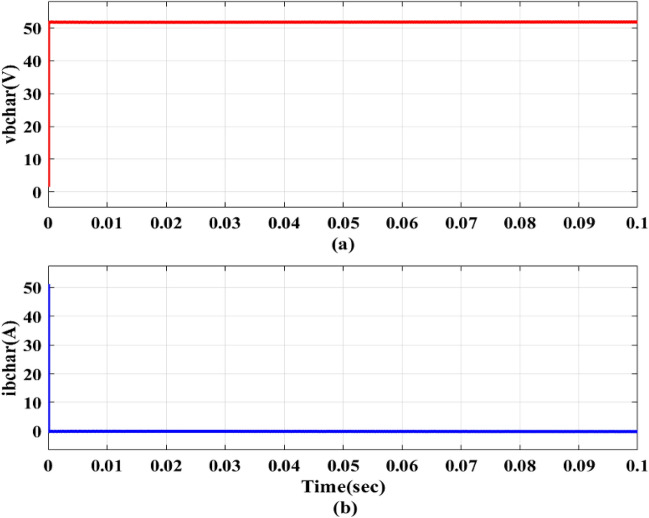
Battery outputs (**a**) v_bchar_ (**b**) i_bchar_.

The EVB is charged by the combination of PV and DC–DC converter contribute 1.12 kW for charging. This process involves monitoring *v*_*pv*_,* i*_*pv*_ and *P*_*pv*_ as depicted in Fig. 13a–c. Meanwhile, the EVB’s operating parameters, *v*_*bchar*_ and *i*_*bchar*_, are maintained at 51.86 V and − 32.88A, respectively, indicating a maximum charging current of 32.88A. The negative sign denotes that current entered EVB, aligning with expectations illustrated in Fig. 14a,b.

**Figure 15:**
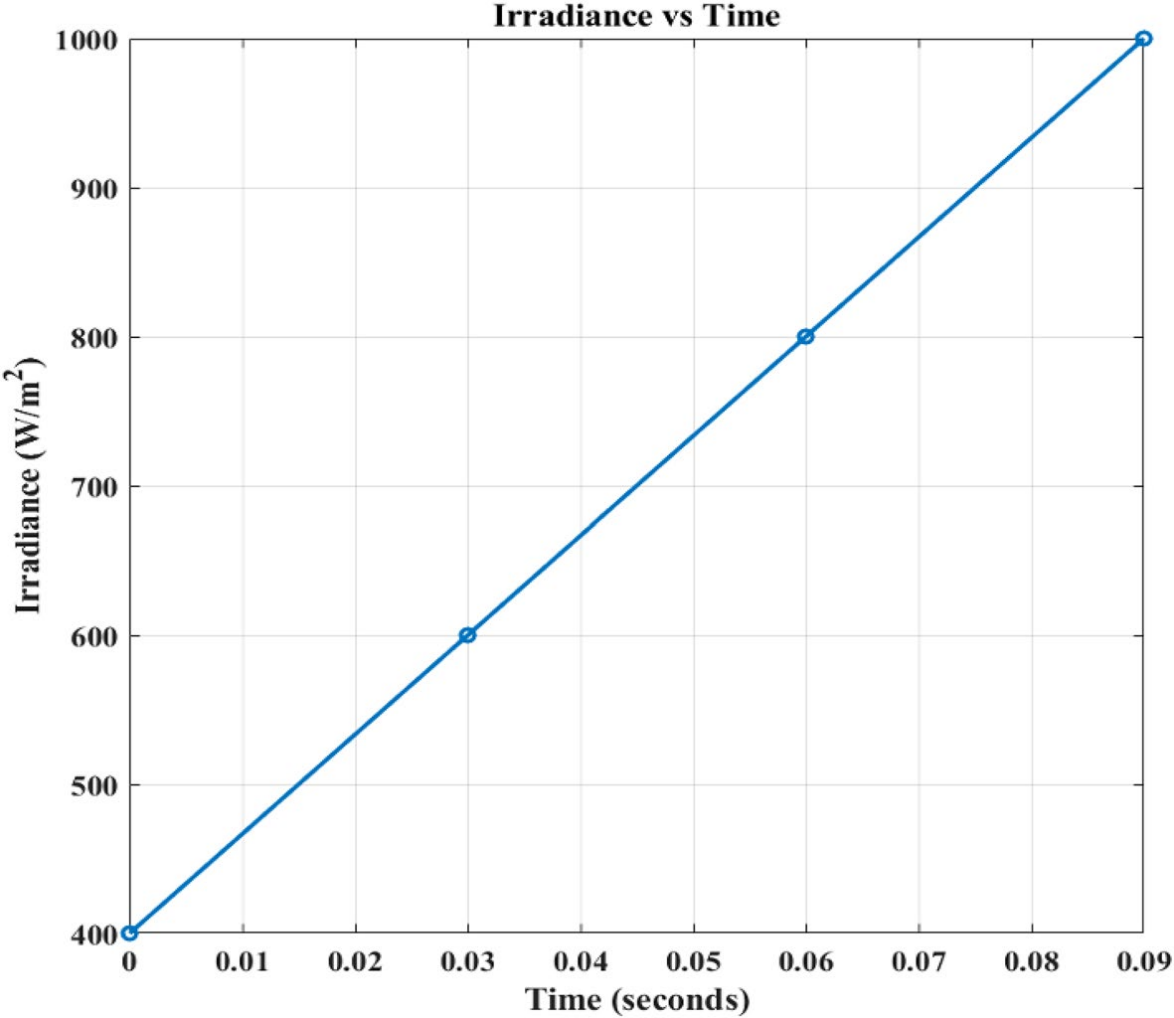
Irradiance variation.

**Figure 16:**
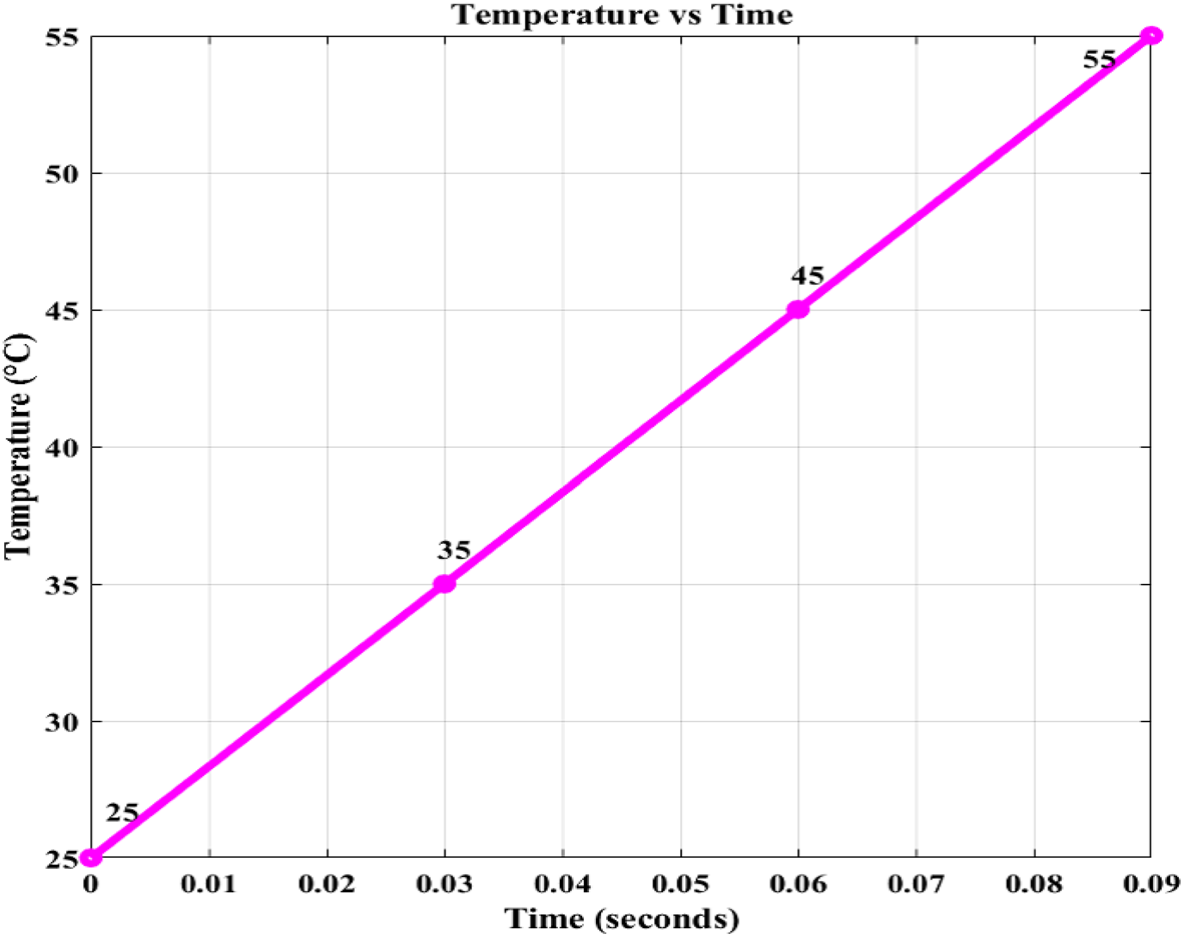
Temperature Variation.

**Figure 17:**
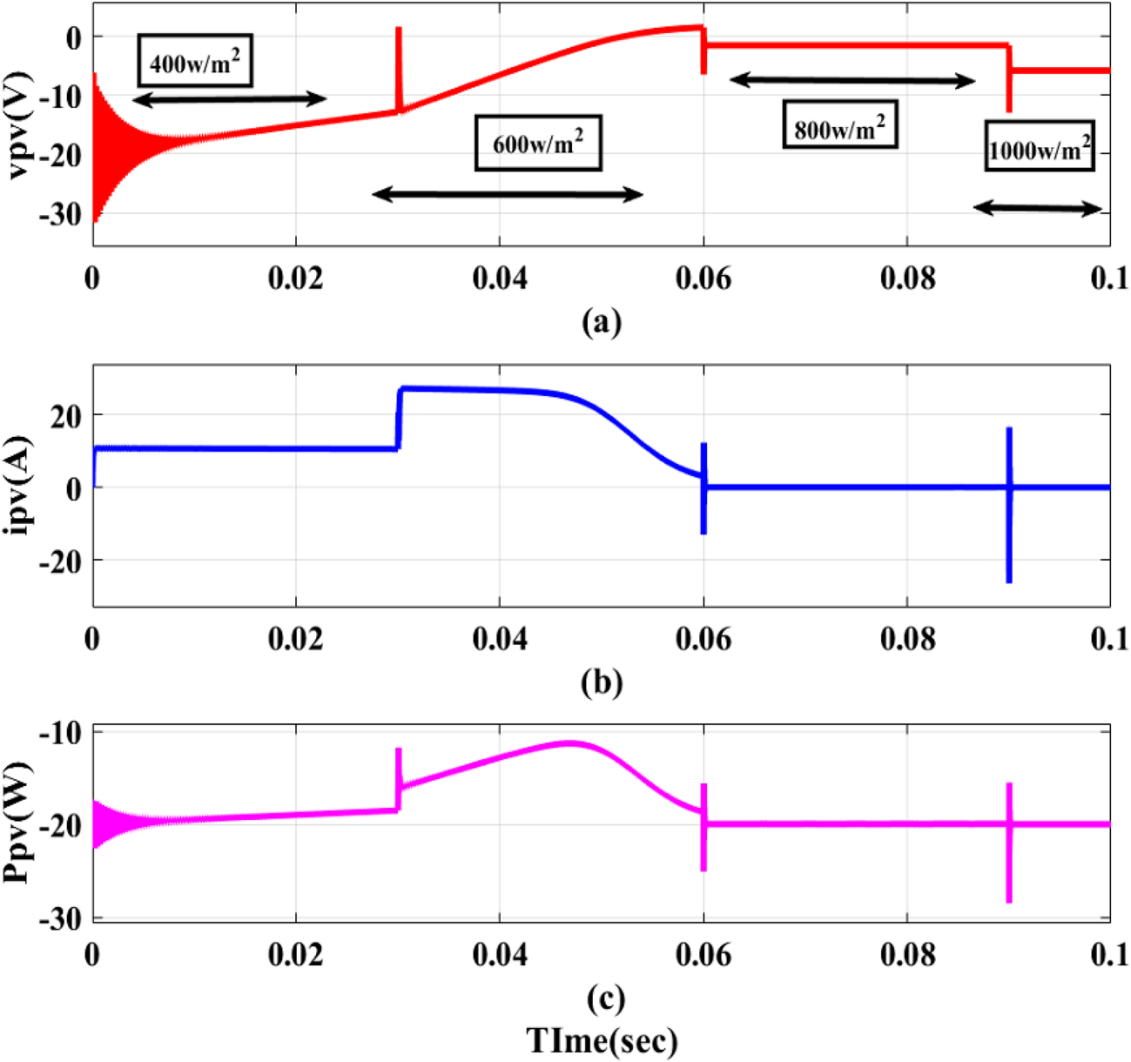
PV outputs with Variation of irradiance and temperature.

Figures 15 and 16 shows the variation in irradaince and temperature. Figure 17 illustrates four alternative states in which the intensity varies with 400/m^2^, 600/m^2^, 800/m^2^, and 1000/m^2^. The temperature varies with 25 degrees and the PV outputs are *v*_*pv*_, *i*_*pv*_, and *p*_*pv*_. 25 °C, 35 °C, 45 °C, 55 °C. All of these were fed to three EVB. Regardless of variances, each battery displays *i*_*bchar*_ of -32.88A and *v*_*bchar*_ of 51.86 V. The negative sign before the current measurement means that the current is flowing into the battery.

Utilizing the M-LRMRAC technique for second-order solar PV systems yields remarkably MPP attainment across varying irradiance and temperature conditions, often within fractions of a second. The convergence time for these specific parameters is detailed in Table 2.

**Table 2:** Convergence time for different techniques with M-LRMRAC and P&O.

Irradiance (W/m^2^)	Temperature (°C)	M-LRMRAC (s)	P&O (s)
1000	25	0.54	10.1
1000	30	0.54	10.2
1000	35	0.54	10.3
1000	40	0.54	10.4
1000	45	0.54	10.5
800	25	0.47	11.9
800	30	0.47	11.8
800	35	0.47	11.7
800	40	0.47	11.6
800	45	0.47	11.5
600	25	0.39	13.9
600	30	0.39	13.8
600	35	0.39	13.7
600	40	0.39	13.6
600	45	0.39	13.5
500	25	0.29	14.9
500	30	0.29	14.8
500	35	0.29	14.7
500	40	0.29	14.6
500	45	0.29	14.5
400	25	0.09	15.9
400	30	0.09	15.8
400	35	0.09	15.7
400	40	0.09	15.6
400	45	0.09	15.5

Table 2 reveals that for a consistent MPP at 37.65 V and 8.9A, yielding 335W, the M-LRMRAC method out- performs the P&O technique in achieving rapid MPP. For instance, under specific conditions such as 1000 w/m^2^, irradiation and 25 °C temperature, the M-LRMRAC technique attains MPP within just 0.54 s, while the P&O method takes 10.1 s. A detailed comparison for the proposed system along with existing topologies present in Table3.

**Table 3:** Comparison of different topologies with the proposed system.

References	^12^	^50^	^53^	Proposed
System Integration	EV integrated converter	EV charging station with solar assistance at level 2	DG, grid-based CS, solar PV, and battery storage	MPPT PV system with PFC and DC–DC for EV charging
Power Sources	Utility grid and solar PV	Solar PV	Solar PV, Battery, Diesel Generator, Grid	Utility grid and solar PV
Converter Types	Isolated SEPIC for charging, Boost, Buck	Buck Converter for charging	SEPIC, Boost, Buck	Boost Converter for MPPT with PFC and DC–DC for EV charging
MPPT Technique	P & O	P & O	P & O	M-LRMRAC
Tracking Speed (MPP Capture Time)	Not specified	Not specified	Not specified	0.54 s
MPPT under dynamic condition of PV	Oscillation present at MPP	Oscillation present at MPP	Oscillation present at MPP	Oscillation is absent at MPP

## Conclusion

Finally, this study presents a novel strategy for integrating photovoltaic systems with AC–DC Power Factor Correction for efficient and reliable electric vehicle charging. The PFC system’s bi-directional power flow regulation optimizes resource utilization and increases EV battery capacity under variable environmental circumstances. The Modified Lyapunov-Based Robust Model Reference Adaptive Controller (M-LRMRAC) implementation for real-time Maximum Power Point Tracking (MPPT) improves MPP convergence speed, exceeding traditional methods such as Perturb and Observe (P&O). Furthermore, the benefits of the suggested approach go beyond efficient MPPT. The EV charging process shows a significant improvement in power quality with Unity Power Factor (UPF) operating and reduced Total Harmonic Distortion (THD). This holistic strategy is in line with the goals of efficient energy use and sustainable transportation networks. PV system integration with AC–DC PFC, combined with the M-LRMRAC method, represents an appealing possibility for expanding clean and efficient EV charging technology. This study contributes to the realization of a cleaner and more sustainable transportation landscape, so assisting the global shift towards lower carbon emissions and better energy efficiency.

Since a result of this study's solid foundation for effective EV charging, various options for future research arise. Additional studies into advanced control techniques, such as AI-based controllers, could improve MPPT precision and system efficiency as a whole. Explore strategies for smoothly integrating the EV charging infrastructure with the grid, taking into account load management, smart grid technology, and bidirectional energy flow. Investigate the use of energy storage devices when combined with the proposed system to handle energy fluctuations, improve charging flexibility, and improve grid interaction. Real-world testing and validation should be carried out to evaluate the efficacy and efficacy of the proposed integrated EV charging system in practical circumstances. Conduct thorough economic evaluations to determine the economic value of the suggested method in contrast to traditional EV charging alternatives. Look into the incorporation of Vehicle-to-Grid (V2G) features, which let EVs send energy back to the grid during times of high demand. To calculate the decrease in carbon emissions brought on by the integrated PV-based EV charging strategy, do an environmental impact assessment. By exploring these topics, future research can help to improve and enhance the integration of renewable energy sources, cutting-edge control techniques, and grid interconnections within the context of EV charging, eventually leading to the development of more sustainable and effective transportation systems.

## Data Availability

The datasets used and/or analysed during the current study available from the corresponding author on reasonable request.

## List of symbols

i_s_: Supply current
*v*_*s*_: Supply voltage
*F′*(*D*): Connection between PV and D
*δI*_*L*_: Ripple current in the L
*γ*: Adaptation gain
*d*(*s*): Small signal variation D
*θ*_1_, *θ*_2_, *θ*_3_: Controlled parameters of plant
*a*_*m*_, *k*_*m*_, *b*_*m*_: Reference model parameters
*b*_*p*_, *a*_*p*_, *k*_*p*_: Plant parameters
*f*_*s*_: Switching frequency
*i*_*bB*_: Battery current
*i*_*bchar*_: Charge current of the battery
*i*_*b*_: Battery current
*i*_*Lp*_, *i*_*Ln*_: Inductor currents of PFC converter
*I*_*sc*_: Short circuit current
*k*_*m*_: Positive gain
*N*_*pe*_: Number of parallel strings
*N*_*se*_: Number of series strings
*P*_*max*_: Maximum power
*R*_*i*_, *C*_*in*_: Input resistance and input capacitor of so lar boost converter
$V_{{b_{{{\text{char}}}} }}$: Charge voltage of the battery
*v*_*b*_: Battery voltage
*V*_*oc*_: Open circuit voltage
*v*_*pv*_, *i*_*pv*_: PV array voltage and current
*v*_*ref*_: Reference voltage
AGMVC: Adaptive generalized maximum versoria criterion
ANFIS: Adaptive neuro-fuzzy inference system
AQAB: Asymmetrical quadruple active bridge
BES: Battery energy storage
BLDC: Brushless DC
CCM: Continuous conduction mode
D: Duty cycle
DAB: Dual active bridge
DAB SRC: Dual active bridge series resonant converter
DG: Diesel generator
DSP: Digital signal processing
ESS: Energy storage system
ESU: Energy storage unit
EV: Electric vehicle
EVB: Electric vehicle battery
FLC: Fuzzy logic control
ICEV: Internal combustion electric vehicle
INC: Incremental conductance
L: Input inductor in boost converter
LV, MV: Low voltage, medium voltage
M-LRMRAC: Model Lyapunov predicated robust model reference adaptive controller
MPP: Maximum power point
MRAC: Model reference adaptive control
MPPT: Maximum power point tracking
P&O: Perturb and observe
PCC: Point of common coupling
PEI: Power electronic interface
PEV: Pug in electric vehicle
PF: Power factor
PFC: Power factor correction
PHEV: Pug in hybrid electric vehicle
PID: Proportional-integral-derivative
PQ: Power quality
PRN: Propulsion
PV: Photovoltaic
PWM: Pulse width modulation
QAB: Quadruple active bridge
r(t): Input to the entire M-LRMRAC controller
RBG: Regenerative braking
RV: Road vehicles
SMG: Smart micro grid
SOC: State of charge
TF: Transfer function
THD: Total Harmonic distortion
UVT: Unit vector template
V_dc_: Dc link voltage
VSC: Voltage source converter
ZVS: Zero voltage switching
